# Identification of adipose tissue transcriptomic memory of anorexia nervosa

**DOI:** 10.1186/s10020-023-00705-7

**Published:** 2023-08-15

**Authors:** Rizaldy C. Zapata, Chanond A. Nasamran, Daisy R. Chilin-Fuentes, Stephanie C. Dulawa, Olivia Osborn

**Affiliations:** 1 Division of Endocrinology and Metabolism, School of Medicine, University of California San Diego, San Diego, USA; 2 Center for Computational Biology & Bioinformatics, School of Medicine, University of California San Diego, San Diego, USA; 3 Department of Psychiatry, School of Medicine, University of California San Diego, La Jolla, 92093 San Diego, CA USA

**Keywords:** Anorexia nervosa, Undernutrition, Transcriptomic memory, Adipose tissue, Calm2, Vps13d

## Abstract

**Background:**

Anorexia nervosa (AN) is a complex debilitating disease characterized by intense fear of weight gain and excessive exercise. It is the deadliest of any psychiatric disorder with a high rate of recidivism, yet its pathophysiology is unclear. The Activity-Based Anorexia (ABA) paradigm is a widely accepted mouse model of AN that recapitulates hypophagia and hyperactivity despite reduced body weight, however, not the chronicity.

**Methods:**

Here, we modified the prototypical ABA paradigm to increase the time to lose 25% of baseline body weight from less than 7 days to more than 2 weeks. We used this paradigm to identify persistently altered genes after weight restoration that represent a transcriptomic memory of under-nutrition and may contribute to AN relapse using RNA sequencing. We focused on adipose tissue as it was identified as a major location of transcriptomic memory of over-nutririon.

**Results:**

We identified 300 dysregulated genes that were refractory to weight restroration after ABA, including *Calm2* and *Vps13d*, which could be potential global regulators of transcriptomic memory in both chronic over- and under-nutrition.

**Conclusion:**

We demonstrated the presence of peristent changes in the adipose tissue transcriptome in the ABA mice after weight restoration. Despite being on the opposite spectrum of weight perturbations, majority of the transcriptomic memory genes of under- and over-nutrition did not overlap, suggestive of the different mechanisms involved in these extreme nutritional statuses.

**Supplementary Information:**

The online version contains supplementary material available at 10.1186/s10020-023-00705-7.

## Introduction

Anorexia nervosa (AN) is a complex and serious disorder characterized by intense fear of weight gain and a disturbed body image, which encourage severe food restriction or other weight loss behaviors such as excessive physical activity. AN affects up to 4% of women and 0.3% of men (Eeden et al. 2021) and has the highest mortality rate of any psychiatric disorder. AN is notoriously difficult to treat, with patients showing a relentless pursuit to thinness by chronically restriciting food consumption and compulsively exercising. Alternations in neuronal networks controlling the homeostatic mechanisms regulating food intake and energy expenditure, as well as non-homeostatic mechanisms driven by affective and cognitive processes interact to drive or suppress feeding (Rossi and Stuber 2018). In addition, there is a high degree of crosstalk between both peripheral and central signals influencing food intake (Gupta et al. 2020; Torres-Fuentes et al. 2017).

Chronic metabolic diseases have been postulated to induce irreversible physiologic and cellular changes which encourage the development of new body weight set points (Speakman et al. 2011). For example, obese individuals that lose weight struggle to maintain a reduced body weight over time due to persistent molecular changes, that affect energy expenditure (Goldsmith et al. 2010; Rosenbaum et al. 2003, 2005) and appetite regulation (Sumithran et al. 2011; Doucet et al. 2000), which encourage weight re-gain. In rodent models, these persistent changes encompass the metabolome (Hernandez-Carretero et al. 2018), and transcriptome (Fischer et al. 2018; Zapata et al. 2022), particularly in the adipose tissue. Conversely, successful realimentation in AN results in weight restoration, but recidivism is common, and the rate of relapse is estimated to be ranging from 30 to 50% (Berends et al. 2016; Carter et al. 2012; Pike 1998). However, the underlying mechanisms driving AN recidivism is under studied.

The rodent Activity-Based Anorexia (ABA) model induces some of the core features of AN, including reduced food intake and hyperactivity despite profound weight loss (Zhang and Dulawa 2021). In the ABA paradigm, rodents are singly housed with running wheels and exposed to scheduled feeding. Under these conditions, animals develop compulsive wheel-running and decreased calorie intake despite extreme weight loss. Interestingly, female rodents develop greater hyperactivity and lose body weight faster than males during ABA, paralleling the female preponderance in AN. However, a limitation of this paradigm is that rodents often lose 25% of their body weight, termed “dropout”, from the paradigm very rapidly. In a typical experiment, all mice dropout in less than a week after the start of the experiment (Beeler et al. 2021; Ho et al. 2016; Schalla and Stengel 2019). Thus, the chronicity of most cases of AN in humans has not been adequately modeled. Here, we modified the prototypical ABA paradigm to mimic the persistency of AN and identified potential adipose-derived mediators of AN recidivism.

## Materials and methods

### Modified activity-based anorexia (ABA)

**Fig. 1:**
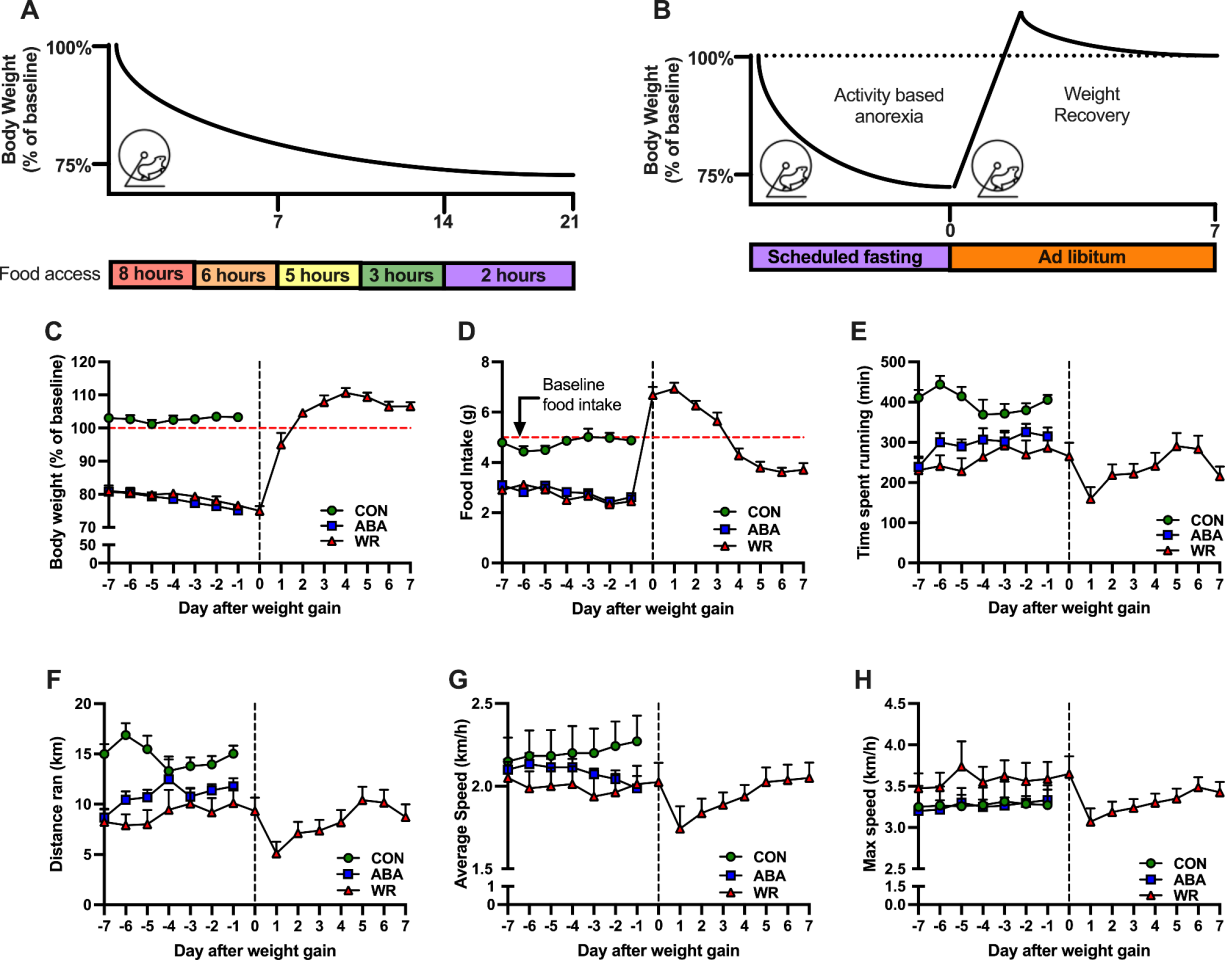
Modified Activity-Based Anorexia model in mice for the study of recidivsm. **(A)** Diagram of the progressive reduction in food access duration during ABA. **(B)** Study timeline of ABA with weight restoration period. **(C)** Body weight changes, as percent of of baseline, **(D)** Food intake, **(E)** Time spent running, **(F)** Distance ran, **(G)** Average and **(H)** Maximum running speed of CON (n = 8), ABA (n = 8) and WR (n = 8) mice. Data was expressed as mean SEM and analyzed with two-tailed repeated measures ANOVA followed by two-stage linear step-up procedure of Benjamini, Kreiger and Yekutieli with an FDR of 0.1. Significance was set at p < 0.5

The animal care protocol was approved by UCSD IACUC. Nine-week-old female C57BL/6J mice were purchased from The Jackson Laboratory (Catalog #000664, Sacramento, CA) and were acclimated to experimental conditions (12:12 light-dark cycle, 22-25 °C, 40–50% humidity) for 3 days. We utilized 9-week old female mice as this age and gender reflect the higher prevalence of AN in adolescent females compared to males. In addition, C57BL/6 is a commonly used mouse strain in ABA (Robinette et al. 2020; Daimon and Hentges 2022; Miletta et al. 2020; Welch et al. 2019). Mice were singly housed and were adapted to running wheels for 7 days with *ad libitum* normal chow diet and water. Mice were randomized into 3 groups: Control (*ad libitum* food and access to running wheel, n = 8), ABA (scheduled feeding and access to running wheel, n = 8), and Weight Restored (WR, scheduled feeding followed by *ad libitum* feeding after 25% weight loss and access to running wheel, n = 8). ABA and WR mice were subjected to a progressive reduction in the duration of food access during the light period (Fig. 1A**)**. They were fed from 0900 to 1700 (8 h, Day 1–4), until 1500 (6 h, Day 5–7), until 1400 (5 h, Day 8–11), until 1200 (3 h, Day 12–14) and until 1100 (2 h, Day 15 to unitil the lose 25% of their initial body weight). ABA mice were sacrificed upon losing 25% of their baseline body weight. A yoked CON mouse was paired with an ABA mouse such that it was sacrificed when an ABA mouse dropped out of the experiment. WR mice were sacrificed once they regained and stabilized their pre-ABA body weight for 2 days (Fig. 1B**)**. Blood, hypothalamus and gonadal adipose tissues were collected, flash-frozen in liquid nitrogen and stored at -80 °C until analysis. The mouse experiment was performed once by a single investigator (RZ) and no blinding was implemented.

### Quantitative real-time PCR and ELISA

Total RNA was isolated from the hypothalamus and purified using Trizol and alcohol precipitation. RNA concentration and quality were assessed using Nanodrop. cDNA was synthesized from 500 ng of RNA using High-Capacity cDNA transcription kit (Thermo Fisher). qPCR was performed using StepOne Plus (Applied Biosciences). Hypothalamic gene expressions of *Agrp, Npy, Pomc* and *Cart* were normalized to housekeeping *Hprt1* and *Pgk1* (Li et al. 2014). Primer sequences used are listed in Supplementary Table 6. Circulating plasma levels of leptin were measured in plasma by EZML-82 K (Millipore Sigma).

### RNA sequencing

The RNA seq was performed through the Institute of Genomic Medicine, UCSD. Total RNA was isolated from the gonadal WAT and purified using Trizol and commercial isolation kits (Qiagen RNAeasy). The quality of the RNA was assessed using the Tapestation 2200 (Agilent) and all samples passed an RNA Integrity score (RIN) above 7.5 denoting good quality RNA. Libraries were prepared using TruSeq Library prep kits (Illumina) and ran on the NovaSeq S4 (Illumina) to obtain an approximate coverage of 10 million reads per sample. Quality control of the raw fastq files was performed using the software tool FastQC v0.11.8. Sequencing reads were trimmed with Trimmomatic v0.38 and aligned to the mouse genome (GRCm38p6) using the STAR aligner v2.5.1a. Read quantification was performed with RSEM v1.3.0 and the Ensembl release 98 annotation. The R BioConductor packages edgeR and limma were used to implement the limma-voom method for differential expression analysis. In brief, lowly expressed genes—those not having counts per million (cpm) ≥ 1 in at least 5 of the samples—were filtered out and then trimmed mean of M-values (TMM) normalization was applied. The experimental design was modeled upon condition (~ 0 + condition). The voom method was employed to model the mean-variance relationship in the log-cpm values, after which lmFit was used to fit per-gene linear models and empirical Bayes moderation was applied with the eBayes function. Significance was defined by using an adjusted p-value cut-off of 0.05 after multiple testing corrections using a moderated t-statistic in limma. Functional enrichment of the differentially expressed genes was performed using WebGestalt (including GSEA13). To detect genes significantly changed by the ‘AN’ state that did not revert to their normal levels after weight restoration, we compared the DEGs in (ABA vs. CON) to (CON vs. WR) and identified genes that were no different.

### Rodent weight loss model

To identify common persistently changed genes in the adipose tissue during chronic perturbations in body weight, we cross-referenced the refractory genes of ABA with the refractory genes of overnutrition from our previous obesity-focused study (Zapata et al. 2022). In brief, 9–10 week old male C57BL/6J mice (#000664, Jackson Laboratories) were fed with either a 10% (LFD, D12450, Research Diets, New Brunswick, NJ) or 60% fat diet (HFD, D12492). After 9 weeks of HFD, half of the HFD-fed mice where switched to the LFD for 3 weeks to induce ~ 10% weight loss which is sufficient to restore glucose tolerance and insulin sensitivity to levels observed in lean, normal chow fed mice (Hernandez-Carretero et al. 2018; Li et al. 2010). The mice were then sacrificed, gonadal white adipose dissected and fractionated into adipocytes and stromal vascular fraction (SVF) from which RNA was extracted and purified as described above.

### Statistics

All data are expressed as mean ± standard error of mean. Biological replicates are indicated in the figure legends. Statistics were carried out using GraphPad 8.1. Normal distribution was tested using Shapiro-Wilk test prior to proceeding with two tailed one-way ANOVA or a two-way ANOVA with repeated measures followed by post hoc tests involving two-stage linear step-up procedure of Benjamini, Krieger and Yekutieli corrections for multiple comparisons with a false discovery rate of 0.10, whenever applicable. Outliers were detected using the ROUT method. Statistical significance was set at α = 0.05.

## Results

### Modified ABA paradigm increased time to drop-out

We designed our ABA paradigm to prolong the induction of weight loss by progressively decreasing food access time from 8 to 2 h over a period of 3 weeks (Fig. 1A). The mice on ABA lost 25% of their baseline weight on an average 15.5 ± 3.2 days (range 9–20 days), compared to less than 7 days in most published studies (Beeler et al. 2021; Ho et al. 2016; Schalla and Stengel 2019). In addition, despite a 25% weight loss and decreased food intake, ABA mice were hyperactive and did not exhibit any signs of sickness. Due to varying times of dropout and for efficient graphical representation, we adjusted the timeline for each animal to assign the day of 25% weight loss as Day 0 (Fig. 1B). This adjustment made each animal, at a given time, very similar in terms of phenotype as a response to ABA. On average, WR regained the weight back after 7 days of *ad libitum* feeding (Fig. 1C). Overall, ABA mice ate 50% less food compared to CON (2.5 g vs. 5 g). During the first 3–4 days of the weight regain, WR mice ate an average of 6.38 g (5.65–6.94 g). Surprisingly, thereafter, mice ate 25% less food compared to CON levels (3.84 g vs. 5 g) (Fig. 1D). During the scheduled fasting, ABA and WR had decreased running time and distance ran compared to CON while ABA and WR did not have significant difference running time spent (Fig. 1E) and distance ran (Fig. 1F). There were also no difference in average speed (Fig. 1G) and maximum speed (Fig. 1H). In WR group, running time, distance ran, average and maximium running speed during scheduled fasting and ad libitum feeding did not differ.

### Weight-restored ABA mice expressed markers that supress appetite

**Fig. 2:**
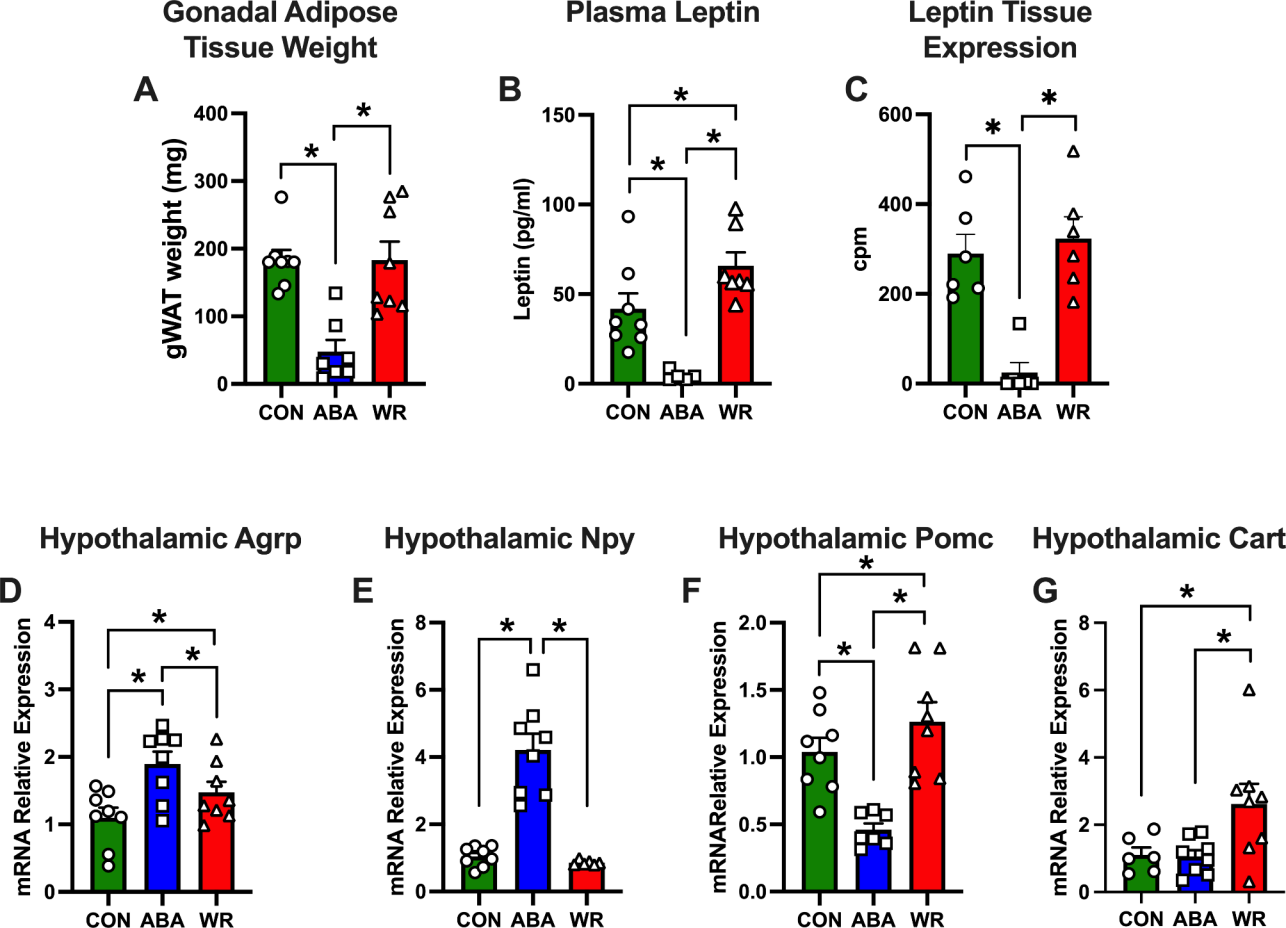
Weight Restored ABA mice expressed markers that supress food intake. **(A)** Gonadal adipose tissue weight (CON: n = 8, ABA: n = 7 and WR: n = 8), **(B)** Leptin plasma concentrations (CON: n = 8, ABA: n = 5 and WR: n = 7), **(C)** Adipose tissue leptin mRNA expression (CON: n = 6, ABA: n = 6 and WR: n = 6. and the hypothalamic expression of the neuropeptides **D.***Agrp*, **E.***Npy*, **F.***Pomc*, and **G.***Cart* in CON (n = 6–8), ABA (n = 7–8) and WR (n = 8) mice. Data was expressed as mean SEM and analyzed with two-tailed one-way ANOVA followed by two-stage linear step-up procedure of Benjamini, Kreiger and Yekutieli with an FDR of 0.1. Significance was set at p < 0.5

Several reports have examined the changes in adipose tissue biology during weight restoration. Waist-to-hip ratio, total trunk fat, visceral and intramuscular adipose tissue were significantly greater in the weight-recovered patients than in the control subjects (Mayer et al. 2005, 2009). It has been reported that there are significantly higher leptin levels in AN patients at target weight than in healthy controls after adjustment for BMI and percent body fat long after weight restoration (Holtkamp et al. 2003, 2004). When AN patients relapse to a lower body weight, it is speculated that high leptin levels represent a counter-regulatory response predisposing to renewed weight loss. Here, we found that adipose tissue expression and plasma concentrations of leptin were reduced during ABA, reflecting the decreased adipose tissue mass. However, despite CON and WR having similar amounts of gonadal adipose tissue, a type of visceral adipose tissue (Fig. 2A), WR mice have increased circulating levels of leptin (Fig. 2B) but without any difference gene expression in the adipose tissue (Fig. 2C) Noteworthy, these data coincided with the decreased food intake observed between day 5–7 of weight regain in WR mice. Thus, our model can be used to examine to role of hyperleptinemia in the hypophagia that happens during AN recidivism.

In the hypothalamus, we also found that the levels of the orexigenic genes *Agrp* (Fig. 2D) and *Npy* (Fig. 2E) increased during ABA, a reflection of the increased hunger. Surprisingly, although *Npy* levels were comparable between CON and WR, *Agrp* levels were increased in WR compared to CON. We also measured the expression of genes that code for anorexigenic peptides. We observed that *Pomc* (Fig. 2F) was reduced in ABA while *Cart* (Fig. 2G) was unchanged. However, both genes were upregulated during WR compared to CON.

### White adipose tissue retain a transcriptomic memory of undernutrition

**Fig. 3:**
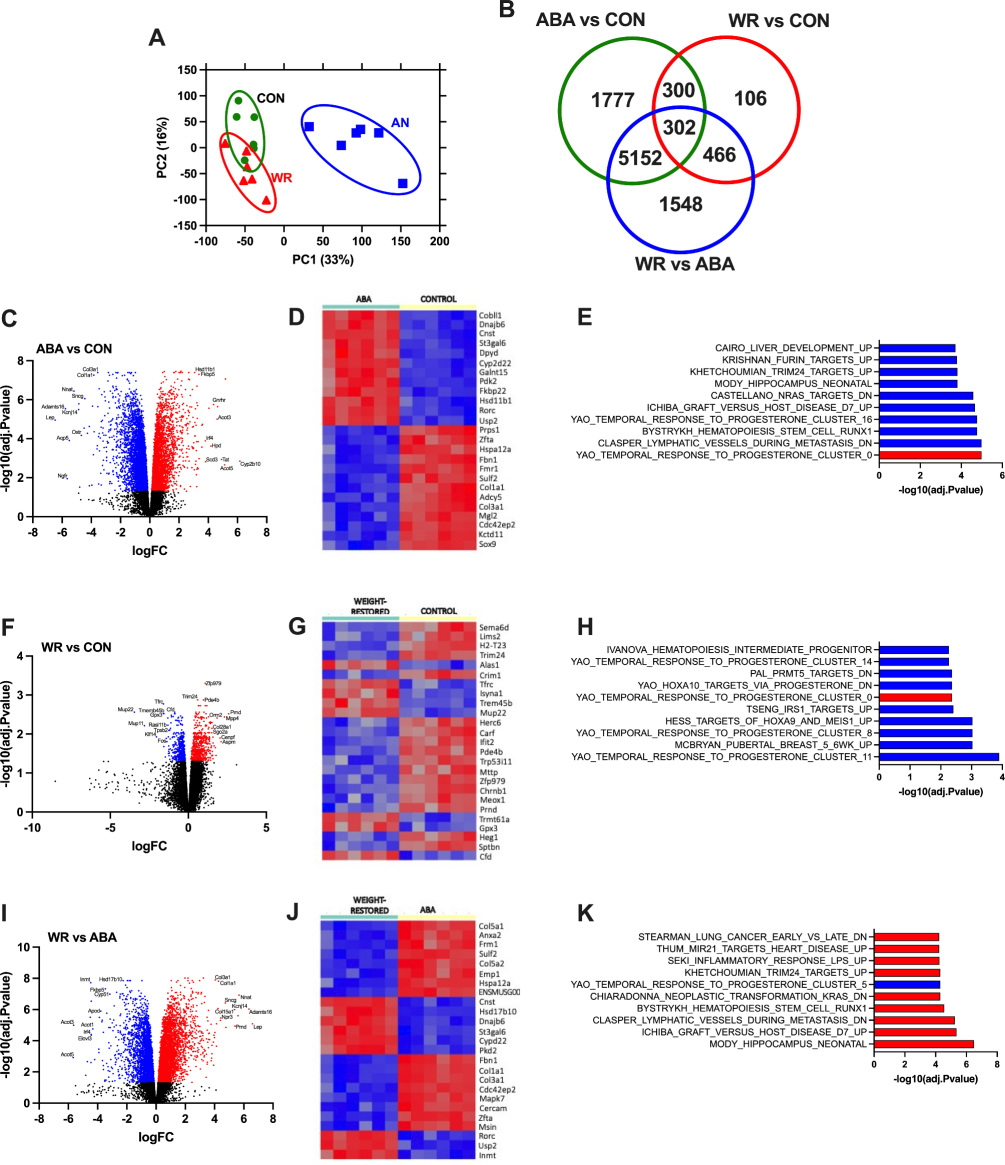
Metabolic memory of AN. **(A)** Principal component analysis of all genes CON (n = 6), ABA (n = 6) and WR (n = 6), **(B)** Venn diagram showing the number of differentially and similarly expressed genes after intersecting the DEGs between ABAvsCON, WRvsCON and WRvsABA. **(C)** Volcano plot of the DEGs, **(D)** Heatmap of the top 25 upregulated and downregulated genes filtered using adjusted *p* value, **(E)** Top 10 enriched pathways using GSEA in ABAvsCON. **(F)** Volcano plot of the DEGs, **(G)** Heatmap of the top 25 upregulated and downregulated genes filtered using adjusted *p* value, **(H)** Top 10 enriched pathways using GSEA in WRvsCON, **(I)** Volcano plot of the DEGs, **(J)** Heatmap of the top 25 upregulated and downregulated genes filtered using adjusted *p* value, **K.** Top 10 enriched pathways using GSEA in WRvsABA. Red points and bars mean upregulated, blue point and bars mean downregulated, black points are not different bewtween the two groups compared

We performed RNA seq studies of the perigonadal white adipose tissue to identify (I) genes that were changed with AN [CON vs. AN], (II) AN genes that were upregulated or downregulated beyond CON levels after WR [CON vs. WR] and (III) persistently changed genes with AN after weight restoration [CON vs. AN] - [CON vs. WR]. Principal component analysis (Fig. 3A) showed that AN transciptomic profile clustered distinctly from CON and WR on PC1 while CON and WR clustered closer to each other on PC1 but not on PC2. Figure 3B illustrates the numbers of genes that are either differentially expressed (DEG) among the 3 groups. Comparing ABA and CON, 6588 protein-coding (3954 were upregulated, 2634 upregulated) DEGs (Supplementary Table 1) were identified, with *Lep* (leptin), *Adamts16* (A disintegrin and metalloproteinase with thrombospondin motifs 16), *Ngfr* (nerve growth factor receptor), *Aqp5* (aquaporin 5), *Nnat* (neuronatin), *Cyp2b10* (cytochrome P450 2B10), *Acot5* (acyl-coenzyme A thioesterase 5), *Tat* (tyrosine aminotransferase), *Acot3* (acyl-coenzyme A thioesterase 3), *Gnrhr* (gonadotropin-releasing hormone receptor) being notable DEGs between these two groups (Fig. 3C). When filtered using adjusted *p*-values, we identified *Cyp2d22* (human ortholog Cyp2d6, cytochrome P450 2D6), *Cnst* (consortin), *Dnajb6* (DnaJ heat shock protein family (Hsp40) member B6*), Rorc* (RAR related orphan receptor C), *Galnt15* (polypeptide N-acetylgalactosaminyltransferase 15), *Kctd11* (potassium channel tetramerization domain containing 11), *Mgl2* (macrophage galactose N-acetyl-galactosamine specific lectin 2), *Col3a1* (collagen type III alpha 1 chain), *Adcy5* (adenylyl cyclase type V), *Cdc42ep2* (Cdc42 effector protein 2) to be top 10 DEGs (Fig. 3D). Gene set enrichment analysis detected the following pathways to be enriched: progesterone signaling, lymphatic vessel during metastasis and hematopoiesis (Fig. 3E).

When WR was compared to CON, despite having similar body weights and adiposity, 503 downregulated and 541 upregulated genes were found (Fig. 3B, Supplementary Table 2). The DEGs *Mup22* (major urinary protein 1*), Mup11* (major urinary protein 11), *Klf14* (Krueppel-like factor 14), *Gpx3* (glutathione peroxidase 3), *Prnd* (prion-like protein doppel), *Mpp4* (MAGUK p55 subfamily member 4), *Aspm* (abnormal spindle-like microcephaly-associated protein homolog), *Cenpf* (centromere protein F) *and Orm2* (alpha-1-acid glycoprotein 2) had the greatest fold change between WR and CON (Fig. 3F). If we screen using adjusted *p*-values, *Zfp979* (zinc finger protein 979), *Trim24* (transcription intermediary factor 1-alpha), *Pde4b* (cAMP-specific 3’,5’-cyclic phosphodiesterase 4B), *Tfrc* (transferrin receptor protein 1*), Ifit2 (interferon-induced protein with tetratricopeptide repeats 2), Sema6d (*semaphorin-6D*), Crim1 (*cysteine-rich motor neuron 1 protein), *Chrnb1* (acetylcholine receptor subunit beta*), Trp53i11 (*tumor protein p53-inducible protein 11*) and Mup22* were identified to be top 10 DEGs (Fig. 3G). In addition, progesterone signaling, pubertal breast, targets of Hoa9 and Meis1 pathways were discovered to be significantly enriched (Fig. 3H).

We then determined ABA genes that did not revert to normal after weight restoration (Supplementary Table 3). We identified *Inmt* (indolethylamine N-methyltransferase), *Chchd10* (coiled-coil-helix-coiled-coil-helix domain-containing protein 10), *Aff2* (AF4/FMR2 family member 2), *Mrgprb1* (Mas-related G-protein coupled receptor member B1), *Fmo1* (flavin-containing monooxygenase 1), *Prnd, Aplnr (apelin receptor), Ptchd4* (patched domain-containing protein 4), *Apln* (apelin), and *Top2a* (DNA topoisomerase 2-alpha) to have the greatest fold change between WR and ABA (Fig. 3F). The top 10 DEGs (when adjusted *p*-value was taken into consideration) included *Fbn1 (*fibrillin-1), *Col5a2* (collagen alpha-2(V) chain), *Emp1* (epithelial membrane protein 1), *Inmt, Msln* (mesothelin), *Ahnak (*desmoyokin*), Fmo1, Kcp* (kielin/chordin-like protein), *Itpripl2* (inositol 1,4,5-trisphosphate receptor-interacting protein-like 2), *and Large1* (LARGE xylosyl- and glucuronyltransferase 1) (Fig. 3G). Gene set enrichment analysis, highlighted pathways for neonatal hippocampus, graft versus host disease, and lymphatic vessels during metastasis to be highly enriched in WR compared to ABA (Fig. 3H).

We then intersected the ABA DEGs with WR DEGs to pinpoint the potential adipose tissue metabolic memory of under-nutrition and we found that 300 genes that were differentially expressed in the AN state and persisted after weight restoration (Fig. 3B, Supplementary Table 5). The genes *Srm* (spermidine synthase), *Rasa3* (Ras GTPase-activating protein 3), *Ear2* (eosinophil cationic protein 2), *Gga2* (ADP-ribosylation factor-binding protein GGA2), *Gnl3l* (guanine-nucleotide binding protein 3-like), *Gabre* (gamma-aminobutyric acid receptor subunit epsilon), *Mrto4* (mRNA turnover protein 4 homolog), *Gps1* (COP9 signalosome complex subunit 1), *Srebf2* (sterol regulatory element binding transcription factor 2), and *Rasal2* (RAS protein activator like 2) were identified to be the top ABA genes that did not every back to ‘normal’levels after weight restoration. Using adjusted *p*-values, we found *Ear2, Gga2, Gabre, Gnl3l, Mrto4, Srm, Rasa3, Gps1, Srebf2*, and *Hspbp1* (Hsp70-binding protein 1) be top 10 persistently dysregulated genes, representing a transcriptomic memory of previous AN state.

### Identification of potential global mediators of chronic weight perturbations

To identify adipose tissue genes that were persistently dysregulated by over- or under-nutrition, we cross-analyzed the RNA seq data from both models of ABA and obesity to determine potential global metabolic memory genes of chronic weight-related perturbations. This comparative analysis identified 5 adipocyte (*Calm2 -* calmodulin-2, *Abcg1 -* ATP-binding cassette sub-family G member 1, *2610203C22Rik, Ucp2 -* uncoupling protein-2, *Vps13d -* vacuolar protein sorting 13D) and 4 SVF (*Slc16a10 -* monocarboxylate transporter 10, *Abcg1, Cfd -* complement factor D, *Aff3 -* AF4/FMR2 family member 3) genes that were persistently changed by former obesity or undernutrition. We then ascertained which genes were altered in opposite directions in these nutritional extremes and found *Calm2* to be increased in obesity but decreased in ABA while *Vps13d* was decreased during obesity yet increased during ABA.

## Discussion

AN is a chronic condition characterized by extreme malnutrition and excessive physical activity with a high rate of relapse. However, the molecular mechanisms that drive AN recidivism are not fully understood and are likely to involve multiple pathways. We hypothesized that a transcriptomic memory of under-nutrition may play an important role in AN recidivism in a similar manner obeserved in defending the higher set point in over-nutrition during obesity. The most common animal model of AN is the ABA paradigm, wherein mice are subjected to scheduled feeding coupled with free wheel-running. However, a caveat of this paradigm, when typically done, is that most mice will lose 25% of their baseline body weight within a few days, which does not mimic the chronicity of the human condition. In these studies, we prolonged the AN induction phase to an average of 15.5 days, which is twice as long compared to the typical ABA paradigm, to mimic the chronicity of the disease in humans and to facilitate an extended time for the mice to effectively establish a transcriptomic memory of undernutrition.

The white adipose tissue (WAT) is a dynamic endocrine tissue that influences whole body energy homeostasis by acting as an energy reservoir as well as secreting hormones, metabolites and cytokines (Choe et al. 2016). During AN, the WAT shrinks as it is being depleted of energy stores which is accompanied by physiologic, transcriptomic and epigenetic adaptations (Xiao et al. 2020). Transcriptional analysis of the WAT from our modified ABA modelled to the identification of 6588 protein-coding genes that were altered during severe undernutrition. Some of the most notable differentially expressed genes in our study have well-established roles in energy metabolism. Leptin (Matsubara et al. 2002), a well-known adipose-derived anorexigenic hormone whose circulating levels are reflective of adipose tissue expansion and contraction, was decreased with ABA and notably increased in obesity. In addition, nerve growth factor (Bulló et al. 2007), which is the ligand for *Ngfr*, was also decreased in ABA but was reported to be increased by 1.4 fold in the plasma in obese women. In contrast, *Cyp2b10*, a member of the Cytochrome P450 superfamily of enzymes, was increased in ABA, whereas its deletion promoted weight gain and adiposity in mice (Heintz et al. 2019). Moreover, the adipose tissue expression of *Acot5* and *Acot3*, which hydrolyze short acyl-CoA esters in aid of peroxisomal beta-oxidation, were both increased in our ABA paradigm and after a 24-hour fasting in mice (Ellis et al. 2015). *Gnrhr*, the receptor for gonadotrophin releasing hormone, was increased in the adipose tissue in our ABA paradigm. However, data indicated that stimulation of Gnrhr increased lipogenesis in adipocytes in vitro, suggesting it may be acting in a compensatory manner to rescue lipogenesis in adipose tissue during ABA (Li et al. 2022). *Nnat* expression was decreased in the adipose tissue in our ABA paradigm in line with other studies whereby differential expression is associated with lipodystrophy (Braun et al. 2020). Furthermore, the deletion of *Nnat*, is associated with partial leptin resistance resulting in hyperphagia, (Millership et al. 2018) also suggesting a potential role of *Nnat* in metabolic regulation. Additional mechanistic studies are required to examine the direct roles of some of these novel DEGs on feeding behavior and body weight regulation.

Despite CON and WR mice having similar body weights and similar gonadal adipose tissue weights, we still found a large number of differentially expressed genes (n = 1044 DEGs) between these groups including genes with important roles in energy balance and adipose tissue biology. A collection of genes had lower expression in the adipose of the WR group compared with CON, including *Gpx3*, *Orm2, Trim24*, *Pde4b*, and *Sema6d*, while expression of *Tfrc* was increased in WR compared to CON. *Gpx3*, which catalyzes the reduction of hydrogen peroxide and lipid peroxides, was decreased in the adipose tissue of insulin resistant and obese patients (Hauffe et al. 2020). *Orm2*, which functions as a major protein carrier in the plasma, when globally knocked-out in mice, increased adipose tissue and body weights (Sun et al. 2016). Whole-body knockout of *Trim24*, an important transcriptional factor for several nuclear receptors, decreased visceral adipose weight (Jiang et al. 2015) and was also decreased in WR compared to CON. Meanwhile, the genetic ablation of *Pde4b*, which catalyzes the hydrolysis of cAMP, reduced body weight and adipose tissue in mice (McDonough et al. 2020) and was decreased in WR compared to CON. Another gene, *Sema6d*, belongs to the semaphorin family of proteins and functions as a signaling molecule and its deletion inhibited M2 macrophage polarization, accompanied by decreased fatty acid uptake and lipid metabolic reprogramming due to downregulation of PPARγ expression (Kang et al. 2018) while WR decreased its expression compared to CON. Adipocyte-specific deletion of *Tfrc*, which is required for the cellular uptake of iron via receptor-mediated endocytosis, decreased body weight and fat mass (Zhang et al. 2021) but was increased in WR compared to CON. We speculate that these transcriptomic adaptations in WR are likely to be part of a complex network of gene interactions that either serve as forward loop or negative feedback mechanisms as the adipose tissue exponentially regains mass during weight restoration after ABA which necessitates further investigations.

It has previosuly been noted that leptin, which signals to the brain to reduce food intake, is significantly increased in AN patients at target weight after weight restoration compared with healthy controls (Holtkamp et al. 2004). When AN patients relapse to a lower body weight, it is speculated that high leptin levels in the circulation represent a counter-regulatory response predisposing to renewed weight loss. Our mouse data is parallel to these observations in humans, signifying that our modified ABA model can be used to study the role of leptin in AN relapse. In future studies, it will be important to establish whether the relative hyperleptinemia observed after weight restoration contributes to hypophagia. Mechanistically, the anorexic effect of leptin is believed to be represented by its action of both the AgRP-expressing neurons (Xu et al. 2018) and POMC-CART-expressing neurons (Balthasar et al. 2004). Deletion of leptin receptors on POMC neurons caused mice to be mildly obese and hyperleptinemic while deletion of leptin receptors in AgRP neurons caused hyperphagia, obesity, hyperglycemia and insulin resistance. We therefore speculate that the combination of increased leptin, and increased *Pomc* and *Cart* during WR drive the hypophagia observed during weight regain from ABA. Moreover, AgRP-expressing neurons have already been implicated in regulating compulsive exercise and survival (Miletta et al. 2020) while POMC-expressing neurons reduced food anticipatory activity in with no significant changes in body weight or food intake (Daimon and Hentges 2022) in ABA mice. Further studies are warranted to examine any persistent effects of AN on the activity of these hypothalamic neurons after weight restoration.

While AN is commonly described as a psychiatric disorder, it is increasingly recognized as a metabo-psychiatric disorder. Large-scale genome-wide association studies found that AN showed significant genetic correlation with both psychiatric disorders and metabolic traits (Duncan et al. 2017; Watson et al. 2019; Yilmaz et al. 2015). Moreover, conditions characterized by chronic over-nutririton, like obesity, and undernutrition, like AN and cachexia, are at the opposite ends of the weight spectrum and might have common pathways that are dysregulated in opposite directions (Foldi et al. 2021; Molocea et al. 2020). This opposing pattern of correlations further highlights that common metabolic pathways might be dysregulated in these extreme states of under- and over-nutrition. We and others have reported significant adipose tissue gene expression changes induced by either obesity or caloric restriction-induced weight loss, that persist after restoration of a normal body weight (Hernandez-Carretero et al. 2018; Zapata et al. 2022; Schmitz et al. 2016; Hahn et al. 2019). In this study, we have identified 300 differentially expressed genes induced by AN that did not revert back to normal levels after weight restoration. Importantly, some of these persistently dysregulated adipose tissue genes identified in the ABA studies have described roles in the pathophysiology of obesity. For example, adipose tissue expression of *Ear2* was decreased by ABA and is also known to be highly expressed in recruited adipose tissue macrophages during obesity (Lumeng et al. 2007). G Protein Pathway Suppressor 1 (*Gps1)* expression was decreased by ABA and previous studies have shown that a downregulation of *Gps1* in adipocytes retarded adipogenesis (Huang et al. 2012). Spermidine synthase (*Srm*) was decreased in the adipose tissue of ABA mice, and the product of this enzyme, spermidine, is known to play a role in lipolysis in visceral fat (Liao et al. 2021) and increased spermidine levels are associated with obesity (Gao et al. 2022). Sterol Regulatory Element Binding Transcription Factor 2 (*Srebf2)*, the master regulator of cholesterol biosynthesis, was decreased in ABA and increased during obesity (Shimano 2009). RAS Protein Activator Like 2 (Rasal2) expression was increased by ABA, while *Rasal2* null mice displayed decreased adiposity (Zhu et al. 2017). In addition, some of the identified persistently dysregulated genes have already proven roles in energy balance regulation. Socs3 (Ueki et al. 2004), Mmp9 (Unal et al. 2010) and Prmt1 (Choi et al. 2021), which had elevated expression levels in obesity that were reversed by weight loss, had decreased expression in ABA and remained decreased in WR.

We hypothesized that there might be common genes that are dysregulated in opposite directions in obesity and AN that might represent global mediators of weight recidivism. Thus, we overlaid the transcriptomic memories of both over- and under-nutrition in the adipose tissues. Interestingly, the majority of the transcriptomic memory genes in these two conditions are mutually exclusive. Only 5 out of 752 adipocyte, and 4 out of 247 SVF transcriptomic memory genes of over-nutrition (Zapata et al. 2022) overlapped with the 300 transcriptomic memory genes of under-nutrition. In addition, despite obesity and AN being on the opposite ends of the weight spectrum, the majority of the common genes were regulated in similar directions. Interestingly, we identified 2 genes, both of which are found in the adipocytes, that could play a universal role in the development of transcriptomic memory of chronic weight perturbations. *Calm2* encodes calmodulin 2, and its expression was increased in obesity but decreased in ABA and was persistently changed in both conditions. *Calm2* plays a role in signal transduction and has been implicated in diabetes where high expression of *Calm2* induced early-onset diabetes or diabetic nephropathy in mice (Epstein et al. 1989; Yuzawa et al. 2008). The second transcriptomic memory gene identified in both data sets was *Vps13d*, which encodes Vacoular Protein Sorting Homolog D, and a frameshift mutation in *Vps13d* is the primary ethiology of Cohen syndrome, a rare genetic disease characterized by trucal obesity as well as developmental delay, mental retardation, microcephaly, and hypotonia (Kolehmainen et al. 2004). Further investigations are warranted to elucidate the roles of these genes in body weight regulation and their potential as therapeutic targets for weight recidivism in both obesity and AN.

Our studies have several limitations. First, our modified ABA model used a relatively short weight restoration period. A longer weight restoration period may have revealed further changes in body weight, which appeared to decrease over time. Furthermore, it would be interesting to determine how long the hypophagic behavior we observed during this period would continue. Second, though the levels of leptin and expression of hypothalamic genes reflect a hypophagic phenotype, further studies are required to prove causation. Third, due to the limited quantity of adipose tissues from ABA mice, we could only conduct a bulk RNA sequencing of the whole adipose tissue. Further studies are needed to determine the cell-type-specific refractory gene expression changes from adipocytes and the immune-cell-containing stromal vascular cells, that are likely to play a dynamic role the transcriptomic memory of ABA and have established roles in metabolic function in obesity (Abete et al. 2006; Caslin et al. 2020). Finally, additional studies are needed to functionally validate the role of the identified DEGs and their role in the regulation of food intake and body weight. Similar to obesity, ABA is likely to also drive persistent changes in the proteome (Geyer et al. 2016), metabolome (Hernandez-Carretero et al. 2018), microbiome (Thaiss et al. 2016) and epigenome (Martínez et al. 2014). For example, we identified several ABA memory genes that potentially play a role in epigenetic regulation of gene expression. *Fbl* (fibrillarin), *Nsun4* (NOP2/Sun RNA methyltransferase 4), *Mettl1* (tRNA (guanine-N(Rosenbaum et al. 2005)-)-methyltransferase), *Hmgn1* (Non-histone chromosomal protein HMG-14) and *Prmt1* (Protein arginine N-methyltransferase 1) were all persistently decreased by ABA while *Brd8* (bromodomain-containing protein 8) was increased by ABA despite weight restoration. Given that most of these genes were downregulated could suggest that their targets are actively being transcribed. Additional future omics-based approaches including adipose tissue ATAC-seq, proteomics and metabolomics will establish a broader picture of the mechanisms that encourage weight recidivism after chronic under-nutrition. The examination of additional metabolic tissues, including muscle, liver and brain should also be performed to determine if they also retain a transcriptomic memory of under-nutrition.

## Conclusions

We were able to design and execute a modified ABA paradigm to model hypophagia seen during AN recidivism. In addition, our mouse model also was able to recapituate the hyperleptinemia observed in weight restored AN patients. Finally, we identified 300 metabolic memory genes of under-nutrition in the adipose tissue which could potentially mediate hypophagia and AN relapse, with *Calm2* and *Vps13d* as potential universal regulators on transcriptomic memory of chronic pertubations in body weight.

### Electronic supplementary material

Below is the link to the electronic supplementary material.

Supplementary Material 1

## Data Availability

All data for this study are reported in this manuscript. The raw, curated and metadata of RNA sequencing are available at GSE229316.

## Abbreviations

ABA: Activity-based anorexia
AN: Anorexia Nervosa
CON: Control mice
DEG: Differentially expressed gene
SVF: Stromal vascular fraction
WR: Weight restored mice
